# Systematic Study on the Self-Assembled Hexagonal Au Voids, Nano-Clusters and Nanoparticles on GaN (0001)

**DOI:** 10.1371/journal.pone.0134637

**Published:** 2015-08-18

**Authors:** Puran Pandey, Mao Sui, Ming-Yu Li, Quanzhen Zhang, Eun-Soo Kim, Jihoon Lee

**Affiliations:** 1 College of Electronics and Information, Kwangwoon University, Nowon-gu Seoul, South Korea; 2 Institute of Nanoscale Science and Engineering, University of Arkansas, Fayetteville, Arkansas, United States of America; Gazi University, TURKEY

## Abstract

Au nano-clusters and nanoparticles (NPs) have been widely utilized in various electronic, optoelectronic, and bio-medical applications due to their great potentials. The size, density and configuration of Au NPs play a vital role in the performance of these devices. In this paper, we present a systematic study on the self-assembled hexagonal Au voids, nano-clusters and NPs fabricated on GaN (0001) by the variation of annealing temperature and deposition amount. At relatively low annealing temperatures between 400 and 600°C, the fabrication of hexagonal shaped Au voids and Au nano-clusters are observed and discussed based on the diffusion limited aggregation model. The size and density of voids and nano-clusters can systematically be controlled. The self-assembled Au NPs are fabricated at comparatively high temperatures from 650 to 800°C based on the Volmer-Weber growth model and also the size and density can be tuned accordingly. The results are symmetrically analyzed and discussed in conjunction with the diffusion theory and thermodynamics by utilizing AFM and SEM images, EDS maps and spectra, FFT power spectra, cross-sectional line-profiles and size and density plots.

## Introduction

**Fig 1:**
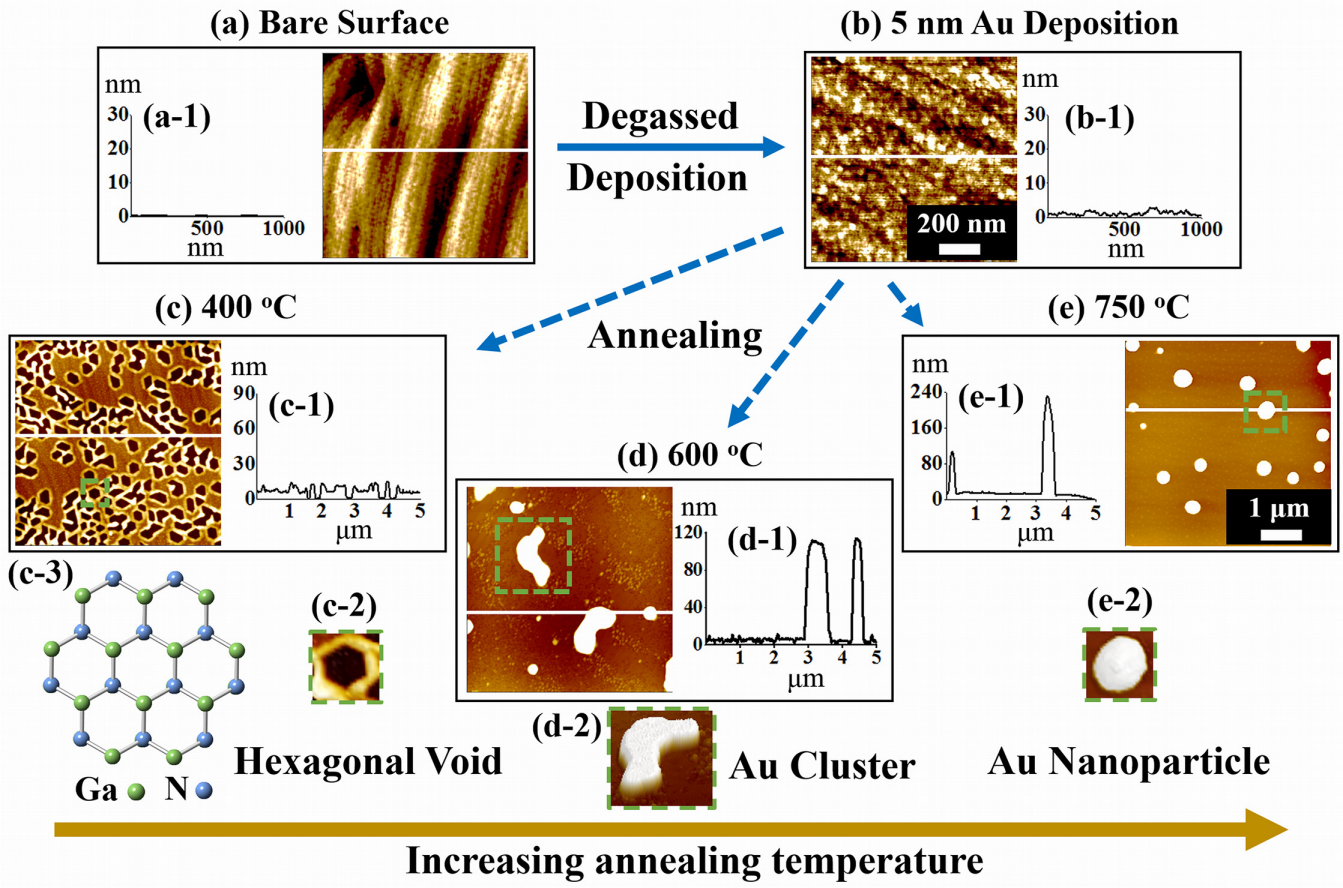
Illustration of the fabrication of hexagonal voids and self-assembled Au nanostructures on GaN (0001) by the variation of annealing temperature. (a) Atomic force microscopy (AFM) top-view of a bare GaN (0001). (b) Surface morphology after the deposition of 5 nm Au. (c) Voids formation after the annealing at 400°C for 300 s. (d) Au nano-clusters observed at 600°C (e) Self-assembled Au NPs fabricated at 750°C. The AFM top-views (a)—(b) are 1 × 1 μm^2^ and that of top-views (c)—(e) are 5 × 5 μm^2^. (a-1)—(e-1) Cross-sectional surface line-profiles obtained from the white lines. (c-2)—(e-2) AFM 3-D views of hexagonal void, Au nano-cluster and Au NPs acquired from the regions denoted with green squares. (c-3) Atomic crystal structure of GaN (0001).

Recently, Au nanoparticles (NPs) have attracted significant research interests due to their potential applications in solar cells,[1,2] memories,[3] sensors,[4] and bio-medical devices[5,6] owing to their localized surface plasmon resonance, increased absorption, enhanced fluorescence and scattering properties.[1–6] The performance of corresponding devices are strongly dependent on the size and density of Au NPs. For example, Au NPs exhibit the localized surface plasmon resonance property that enhances the light absorption so there is significant improvement in the efficiency of solar cells.[1] Comparatively large Au NPs can produce higher power conversion efficiency.[1,2] Meanwhile, small size of Au NPs with the increased density allows improving the turn-on voltages and on/off current ratios in the nanofiber-based memory devices.[3] Also, the arrays of spherical large-scaled Au NPs can be used in the laser fabrication for sensing applications. [4] In addition, Au NPs can enhance the fluorescence intensity so the performance is improved for the detection of DNA in bio-medical devices.[5] Au NPs can also be implemented in cancer diagnosis and photo-thermal therapy due to the enhanced radiative properties such as absorption and scattering by the strong electromagnetic fields on the particle surface.[6] In the meantime, GaN, due to its wide bandgap, strong chemical bonding, high electrical breakdown voltage, high electron mobility and high melting point, has attracted much attention in wide-bandgap optoelectronics and high power devices such as UV light emitting diodes (LEDs),[7,8] photovoltaics,[9] power amplifier,[10] high electron mobility transistors, and etc.[11,12] For the fabrication of GaN nanowires, the size and density of Au NPs can determine the length, width, density and morphology of nanowires.[13] Indeed, Au NPs have been utilized as catalyst for various semiconductor nanowires [14–16] through the vapor liquid solid mechanism with the various growth techniques.[17–19] Although, many applications have been demonstrated utilizing Au NPs on GaN, the systematic study on the size and density of Au NPs on GaN (0001) is still quite deficient and thus, in this work, we investigate the fabrication of self-assembled Au NPs and nanostructures on GaN (0001) by the variation of annealing temperature (T_a_) and deposition amount (DA). Fig 1 shows the illustration of the fabrication of hexagonal Au voids and self-assembled Au nanostructures on GaN (0001) by the variation of annealing temperature. Based on the diffusion limited aggregation model [20], the fabrication of hexagonal shaped Au voids at 400°C and Au nano-clusters at 600°C are successfully demonstrated. The size and density of these nanostructures can systematically be controlled depending upon the annealing temperature. Based on the Volmer-Weber growth model,[21–23] the fabrication of round dome shaped Au NPs is successfully demonstrated at temperatures between 650 and 800°C. The variation in size and density of voids and Au NPs can be systematically analyzed and discussed in terms of the diffusion theory and thermodynamics. We present the AFM and SEM images, EDS maps and spectra, FFT power spectra, cross-sectional line-profiles and size and density plots and the acquired data are systematically analyzed and discussed.

## Materials and Methods

### GaN substrate preparation

The fabrication of self-assembled Au NPs and nanostructures on GaN (0001) by the variation of annealing temperature (T_a_) and deposition amount (DA) was studied with various growth conditions. The GaN (0001) substrates utilized were 10 μm-thick GaN templates grown on sapphire with off-axis ± 0.1° by the Technology and Devices International (TDI, USA). The wafers were treated with the RCA clean. The Raman spectrum of GaN (0001) template is shown in S1 Fig (Supplement). The signal was excited with a CW diode-pumped solid-state (DPPS) laser of a wavelength of 532±1 nm with an output power of 120 mW. The reflectance spectra of GaN (0001) template, UV-Vis-NIR-IR (250–2000 nm), is shown in S2 Fig Deuterium and halogen combined light source was utilized with a CCD for the UV-Vis-NIR (250–1000 nm) regions and an InGaAs photodetector for the IR (1000–2000 nm).

### Fabrication of Au nanostructures

Fig 1 illustrates the fabrication procedure of the self-assembled hexagonal Au voids, nano-clusters and NPs on GaN (0001) by the variation of annealing temperature. Samples were indium-bonded to an Iconel holder and then introduced to a pulsed laser deposition (PLD) chamber for degassing at 700°C for 30 min under 1 × 10^−4^ Torr to remove the contaminants on the surface. Subsequently, various amounts of Au were deposited on samples in an ion-coater chamber at a growth rate of 0.05 nm/s with the ionization current of 3 mA under 1 × 10^−1^ Torr. To clearly observe the annealing temperature effect on the self-assembled Au NPs and nanostructures, the deposition amount of Au as well annealing duration for a series of samples were fixed. For example, the variation of annealing was systematically performed between 400 and 800°C with the constant deposition amount and annealing duration. The annealing procedure was performed in the PLD chamber with the equal ramping rate at 2.3°C/s for each series under 1 × 10^−4^ Torr. After reaching each target temperature, 300 s of dwelling was equally given to all samples. After each growth, the temperature was quenched down immediately to minimize Ostwald ripening. [24, 25] Fig 1(A) shows atomic force microscopy (AFM) top-view of bare surface and Fig 1(B) shows the surface after the deposition of 5 nm Au. The surface morphology of deposited film became a bit bumpy as compared with the bare surface as clearly observed with the cross-sectional line-profiles in Fig 1(a-1) and 1(b-1). Hexagonal Au voids were observed after annealing at 400°C for 300 s as shown in Fig 1(C). Au nano-clusters were observed at 600°C as shown in Fig 1(D). With the increased annealing temperature to 750°C, the self-assembled Au NPs with distinct size were demonstrated as shown in Fig 1(E).

### Characterization of Au nanostructures

Atomic Force Microscopy (AFM) with a non-contact (tapping) mode was employed for the surface morphology characterization. The AFM tips (NSC18/AIBS, μmasch) were with the radius curvature less than 10 nm, height ~ 125 μm and force constant 40 N/m. The scanning was performed at the resonant frequency at ~ 270 kHz in air and same type of tips from a single batch were used for all sample scanning to minimize the tip effect and for the consistency of analysis. The cantilever was Al coated on the back side to increase the reflection of the laser to the position sensitive photo detector (PSPD). The acquired data was analyzed with the XEI (Park System) software, including the AFM top-views, side-views, cross-sectional line-profiles and Fourier filter transform (FFT) power spectra. Also, scanning electron microscopy (SEM) in vacuum was utilized for the larger scale images. For the elemental analysis and phase mapping, energy dispersive X-ray spectroscopy (EDS) (Thermo Fisher Noran System 7) in vacuum was utilized.

## Results and Discussion

**Fig 2:**
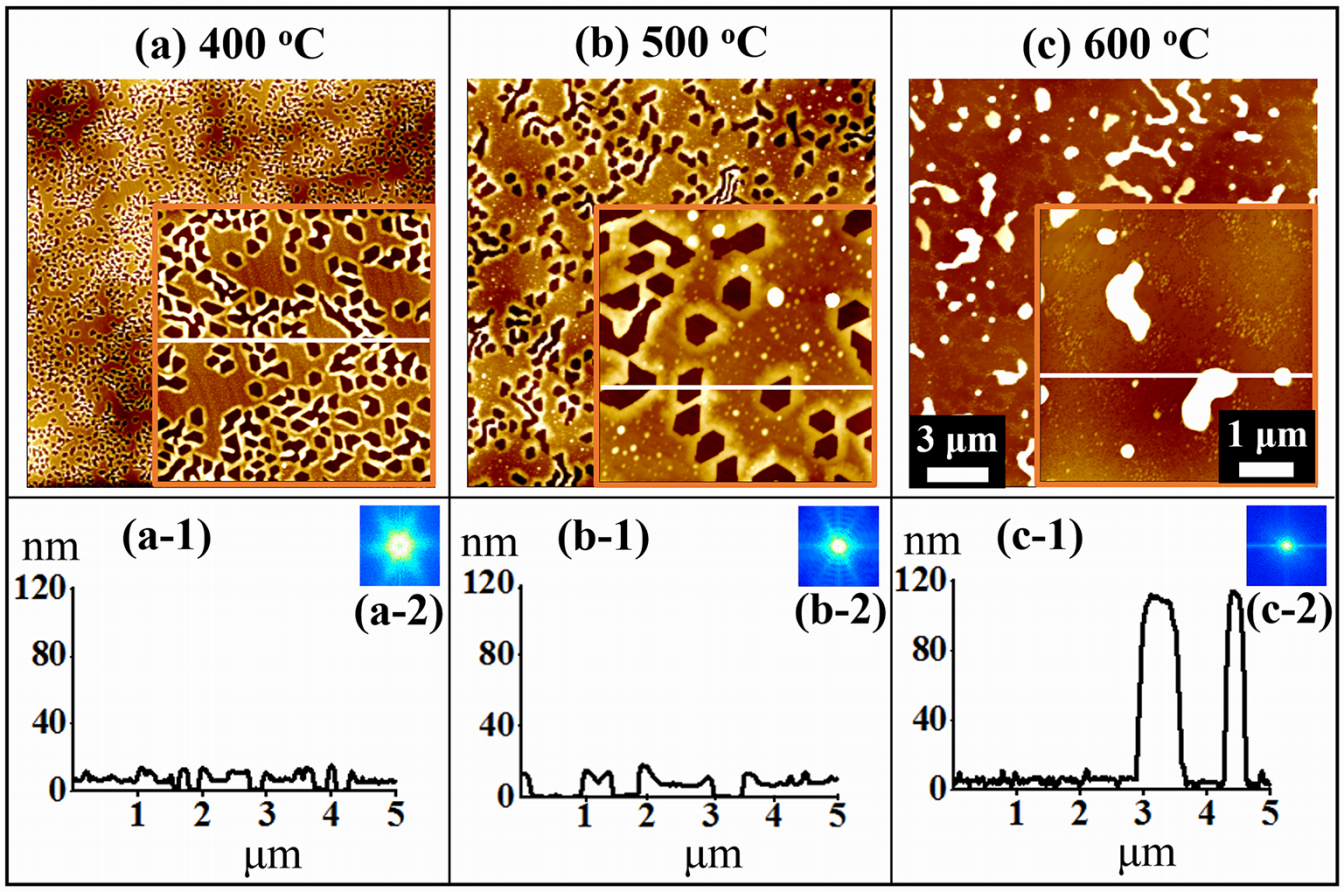
Formation of hexagonal voids and Au nano-clusters on GaN (0001) by the annealing temperature variation between 400 and 600°C for 300 s with 5 nm Au deposition. (a)—(c) AFM top-views of 20 × 20 μm^2^ with insets of 5 × 5 μm^2^. (a-1)—(c-1) Cross-sectional surface line-profiles obtained from the white lines. (a-2)—(c-2) Fourier filter transform (FFT) power spectra.

**Fig 3:**
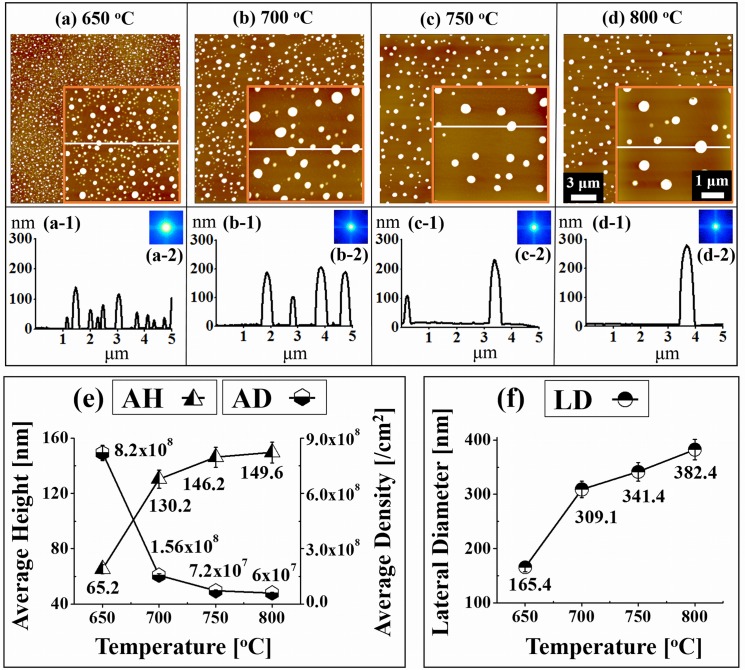
Evolution of self-assembled Au NPs on GaN (0001) by the variation of annealing temperature between 650 and 800°C for 300 s with 5 nm of Au deposition. (a)—(d) AFM top-views (20 × 20 μm^2^) with the insets (5 × 5 μm^2^). (a-1)—(d-1) Cross-sectional surface line-profiles acquired from the insets in (a)—(d). (a-2)—(d-2) FFT power spectra. (e) Plot of average height (AH) and average density (AD). (f) Plot of lateral diameter (LD). Error bars: ± 5%.

Fig 2 shows the fabrication of hexagonal Au voids and nano-clusters on GaN (0001) by the variation of annealing temperature (T_a_) from 400 to 600°C for 300 s with 5 nm Au deposition. The AFM top-views, cross-sectional line profiles and FFT power spectra are presented in Fig 2 and the corresponding 3-D AFM side-views are shown in S3 Fig Between 400 and 600°C of annealing, two distinct phases have been observed: formation of the Au voids and nano-clusters. In general, with the increased T_a_, the size of voids was increased and the density of voids was decreased accordingly. With the initial formation of small voids as clearly shown in Fig 2(A), they are closely packed and connected, and when the voids grew larger in size, the voids showed separation as shown in Fig 2(B). As the T_a_ was further increased, irregular shape of Au nano-clusters were formed as shown in Fig 2(C). The formation of nano-clusters can be due to the increased size of voids by joining nearby ones and as a result, there are isolated regions of Au; namely, the Au nano-clusters. The SEM images in S4(A) and S4(B) Fig show the surface morphologies of the corresponding samples annealed at 500 and 600°C. The fabrication of Au voids, nano-clusters and Au NPs on GaN (0001) can be described on the basis of the thermodynamic relationship between the annealing temperature, surface diffusion coefficient and diffusion length. The surface diffusion coefficient (D_s_) follows a scaling relation of D_s_ ∝ exp (- E_n_ / KT_a_) where E_n_ is the diffusion barrier, K is the Boltzmann constant and T_a_ is the annealing temperature.[26–27] Thus, the surface diffusion coefficient is directly dependent on the annealing temperature. Also, the diffusion length (L_D_) can be obtained from the equation, L_D_ = √ (D_s_ t), where t is the diffusion time. From above two equations, we can settle that the diffusion length is directly dependent upon the annealing temperature (T_a_). In the first instance of annealing at 400°C, Au adatoms can diffuse to aggregate with other Au adatoms due to the enhanced thermal energy. However, there can be a suppression in the aggregation or diffusion of Au adatoms due to a short diffusion length owing to the insufficient or low thermal energy. As a result, hexagonal voids can be formed in Au layer on GaN as shown in Fig 2(A). The cross-sectional surface line-profile in Fig 2(a-1) shows the depth of voids is approximately 8 nm. As shown in Fig 1(c-3), the crystal structure of GaN (0001) is the hexagonal shape with the Ga and N atoms connected each other and thus overall structure over the voids is also the hexagonal. After the annealing at 400°C, the Au atoms can diffuse i.e. the thermal energy activates the Au adatoms such that Au adatoms reassemble accordingly with top-terminated hexagonal close packing (hcp)-GaN crystal structure. Thus, the hexagonal voids formed in Au layer on the surface of GaN (0001) can be the influence of crystal structure of GaN (0001). Similar to this result, Pt and Cu on hexagonal substrates also showed the hexagonal voids or nanoparticles depending upon the growth conditions. For instance, Pt deposition on GaN (0001) annealed at 650°C for 600 s showed Pt layer with hexagonal voids [28] whereas 0.25 monolayer (ML) of Cu on Al_2_O_3_ (0001) annealed at 300°C formed hexagonal Cu nanoparticles.[29] By increasing the annealing temperature to 500°C, the diffusion length was increased and thus the Au adatoms can now aggregate further. And therefore, it can result in the increased size of voids and decreased density as shown in Fig 2(B). The depth of voids was increased to approximately 12 nm observed from the cross-sectional surface line profile in Fig 2(b-1). The metal films of Ag, Cu, Fe, Ag-Ni at low temperature annealing also demonstrated the formation of voids on various substrates. [30–33] Finally, as presented in Fig 2(C), due to the increased diffusion length at 600°C, the Au clusters were fabricated based on the diffusion limited aggregation (DLA) model. [34] The DLA model states that adatoms undergoing random walk due to the Brownian motion aggregate and form Au clusters with the enhanced surface diffusion. The height of Au clusters was drastically increased from only a few nano-meters to over one hundred nano-meters as presented by the cross-sectional line profiles in Fig 2(a-1)–2(c-1). The surface morphology can also be described by the FFT power spectra as shown in Fig 2(a-2)–2(c-2). The FFT power spectrum in Fig 2(a-2) shows the bright hexagonal pattern due to the formation of hexagonal voids and similarly, with the increased size of hexagonal voids, the spectra became smaller and dimmer as shown in Fig 2(b-2). Eventually the FFT power spectra became round and dim when the irregular shaped Au nano-clusters were observed as clearly evidenced by Fig 2(c-2).

Fig 3 shows the evolution of self-assembled Au NPs on GaN (0001) by the variation of T_a_ between 650 to 800°C. The SEM images of corresponding samples between 650 to 800°C are shown in S4(C)–S4(F) Fig With the enhanced thermal energy between 650 and 800°C, the self-assembled Au NPs were successfully fabricated on GaN (0001). In general, as the T_a_ was increased, the dimension of Au NPs was also increased while the density was decreased as clearly shown by the AFM top-views, cross-sectional surface line profiles and FFT power spectra in Fig 3 and the 3-D AFM side-views in S6 Fig Initially, the dome shaped small sized and densely packed Au NPs were observed in Fig 3(A), and when the T_a_ was increased, the Au NPs grew, separated apart and the density was decreased as shown in Fig 3(B). Similarly, the dimension of Au NPs was further increased and the separation got larger and the density was reduced in Fig 3(C) and ultimately Au NPs become much larger in size and the density was further dropped as shown in Fig 3(D). According to the Volmer-Weber growth model, when the binding energy between Au adatoms (E_A_) is greater than that between the Au adatoms and Ga and N atoms (E_G_) (i.e. E_A_ > E_G_), the Au adatoms can strongly be bonded with each other and can form the self-assembled 3-D islands such as Au NPs on GaN with a sufficient diffusion energy provided. [35–38] With the increased annealing temperature, the diffusion length (L_D_) can be increased and as a result the islands can absorb more Au adatoms and the dimension of NPs can be increased while density is decreased. When NPs grow larger, due to the lower surface energy, they have tendency to attract nearby smaller NPs and form even larger NPs until they reach in equilibrium. This is a conventional behavior of metal NPs on various substrates such as Si [34], GaAs [35–37] and SiO_2_.[38] Plots of the average height (AH), average density (AD), and lateral diameter (LD) of the self-assembled Au NPs are presented in Fig 3(E) and 3(F) and their specific values are listed in S1 Table. Initially, at 650°C, the small dome shaped self-assembled Au NPs were observed and the AH, AD and LD were 65.2 nm, 8.2 × 10^8^ cm^-2^ and 165.4 nm, respectively. After increasing the T_a_ to 700°C, the AH was increased by 1.99 times to 130.2 nm, the LD by 1.86 times to 309.1 nm while the AD was dropped by 5.25 times to 1.56 × 10^8^ /cm^2^. With the further increased T_a_ to 750°C, the AH and LD were also increased by 1.12 times to 146.2 nm and by 1.10 times to 341.4 nm, respectively whereas the AD was decreased by 2.11 times to 7.2 × 10^7^ cm^-2^. Finally, at 800°C, the size of Au NPs were further grown and the density kept dropping: the AH to 149.6 nm, LD to 382.4 nm and the AD to 6 × 10^7^ /cm^2^. Overall, the AH and LD were increased by 2.29 and 2.31 whereas the AD was decreased by 13.66 times between 650 and 800°C with 5 nm of Au deposition. Based on the FFT power spectra, the evolution of self-assembled Au NPs with the increased T_a_ can also be observed from Fig 3(a-2)–3(d-2). The FFT power spectra in general were bright round patterns as the shape of NPs. Due to the wide distribution of height of the Au NPs, the FFT spectrum is much larger in Fig 3(a-2) and at increased T_a_ between 700 and 800°C, the FFT spectra appeared to be quite similar indicating similar magnitude of uniformity.

**Fig 4:**
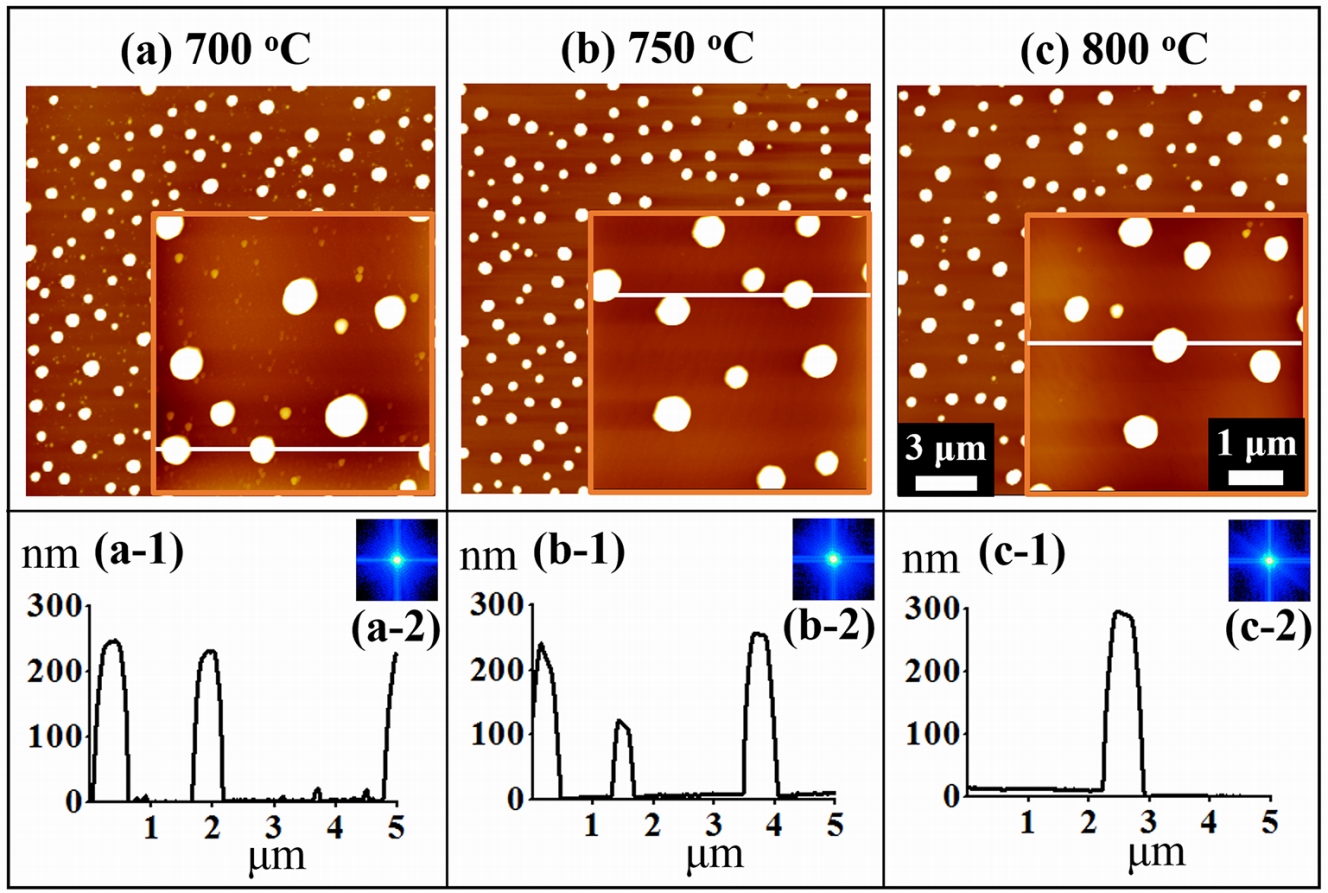
Self-assembled Au NPs with an increased Au deposition amount of 10 nm on GaN (0001) between 700 and 800°C for 300 s. (a)—(c) AFM top-views of 20 × 20 μm^2^ containing smaller scale AFM top-view insets of 5 × 5 μm^2^. (a-1)—(c-1) Cross-sectional surface line-profiles obtained from the white lines. (a-2)—(c-2) FFT power spectra.

**Fig 5:**
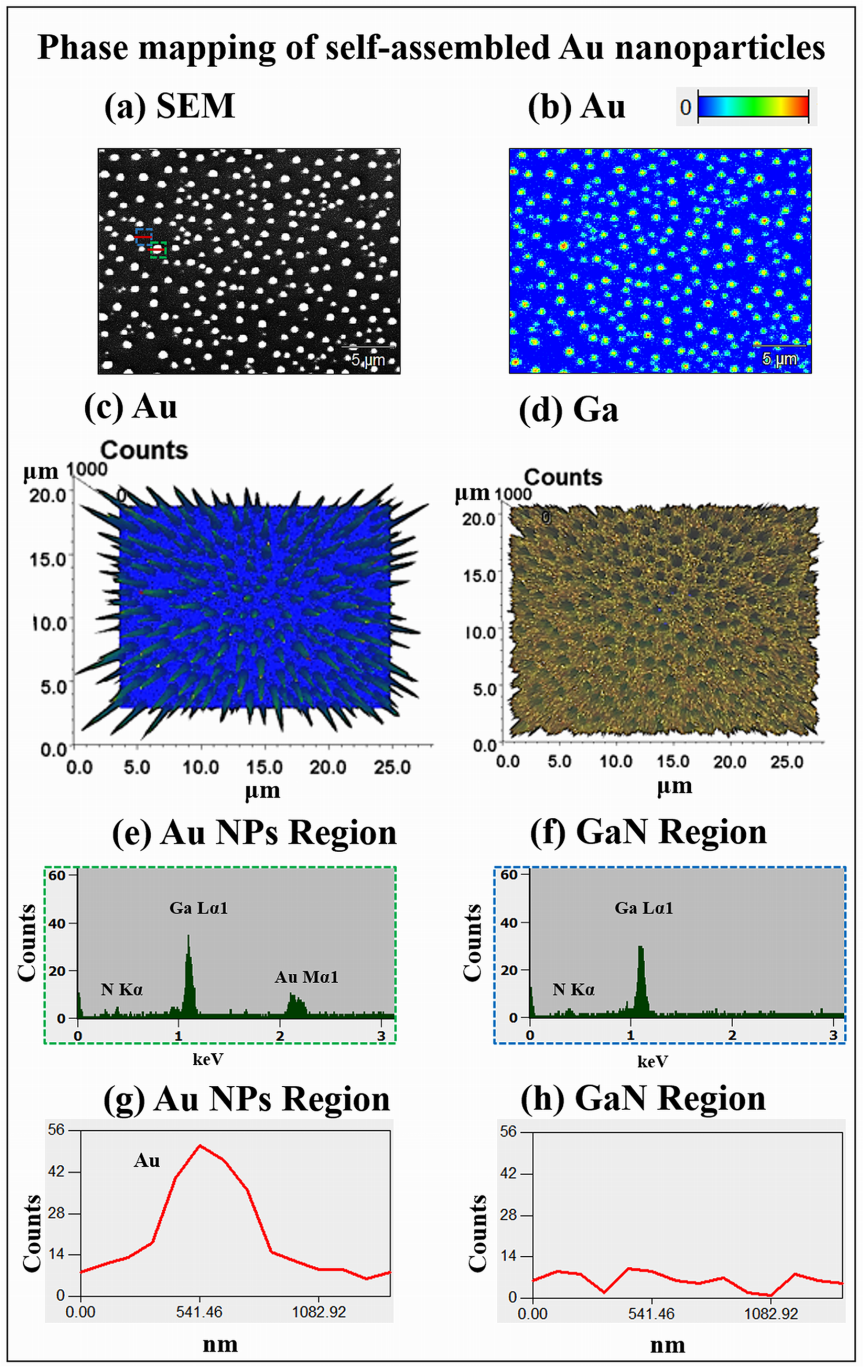
Energy dispersive X-ray spectroscopy (EDS) maps of self-assembled Au NPs with 10 nm of Au deposition annealed at 750°C for 300 s. (a) SEM image. (b) 2-D phase maps of Au. (c)–(d) Au and Ga compositional maps of 3-D top-view. (e)–(f) EDS spectra of particular region marked with the green and blue square in (a). (g)–(h) EDS line-profiles acquired from the red lines in specific region in (a).

Fig 4 shows the self-assembled Au NPs on GaN (0001) with 10 nm of Au deposition by a control of T_a_ between 700 to 800°C for 300 s. Similarly, the AH and LD of Au NPs were increased with the increased T_a_ while the AD was gradually decreased. At first, at 700°C of annealing, the Au NPs showed dome shaped uniform size in Fig 4(A). With 750°C of annealing, the Au NPs grew slightly larger and the density was decreased as shown in Fig 4(B) and eventually, Au NPs grew further and the density kept decreasing in Fig 4(C). The size evolution of self-assembled Au NPs is clearly shown by the cross-sectional surface line profiles in Fig 4(a-1)–4(c-1). The FFT power spectra in Fig 4(a-2)–4(c-2) shows the self-assembled Au NPs morphological evolution with the increased annealing temperature such that the FFT spectra only slightly change in size and brightness due to small change in height distribution and uniformity of the Au NPs. Fig 5 shows the EDS phase maps, spectra and line-profiles of self-assembled Au NPs with the 10 nm Au deposition annealed at 750°C for 300 s. The morphology of self-assembled Au NPs is shown by the SEM image in Fig 5(A) and the corresponding 2-D phase map of Au is presented in Fig 5(B). The 3-D top-views of Au and Ga compositional maps are shown in Fig 5(C) and 5(D). The 2-D phase of Au shows the Au concentration level depending on the colors. The red colored region has highest concentration of Au and the yellow shows slightly low concentration and similarly the green denotes further lower Au. Finally, the blue region shows where Au is not present or minor. As clearly shown in the 3-D top views of Au map, the peaks represent the Au counts whereas remaining region are Ga while in 3-D top views of Ga map, the holes denote the Au and remaining portion shows Ga. The concentration of Au in particular regions can be illustrated by the EDS spectra and line-profiles, as shown in Fig 5(E)–5(H). For instance, the Au peak at Au NPs region in EDS spectra and line-profile shows high counts than that of region without NPs.

**Fig 6:**
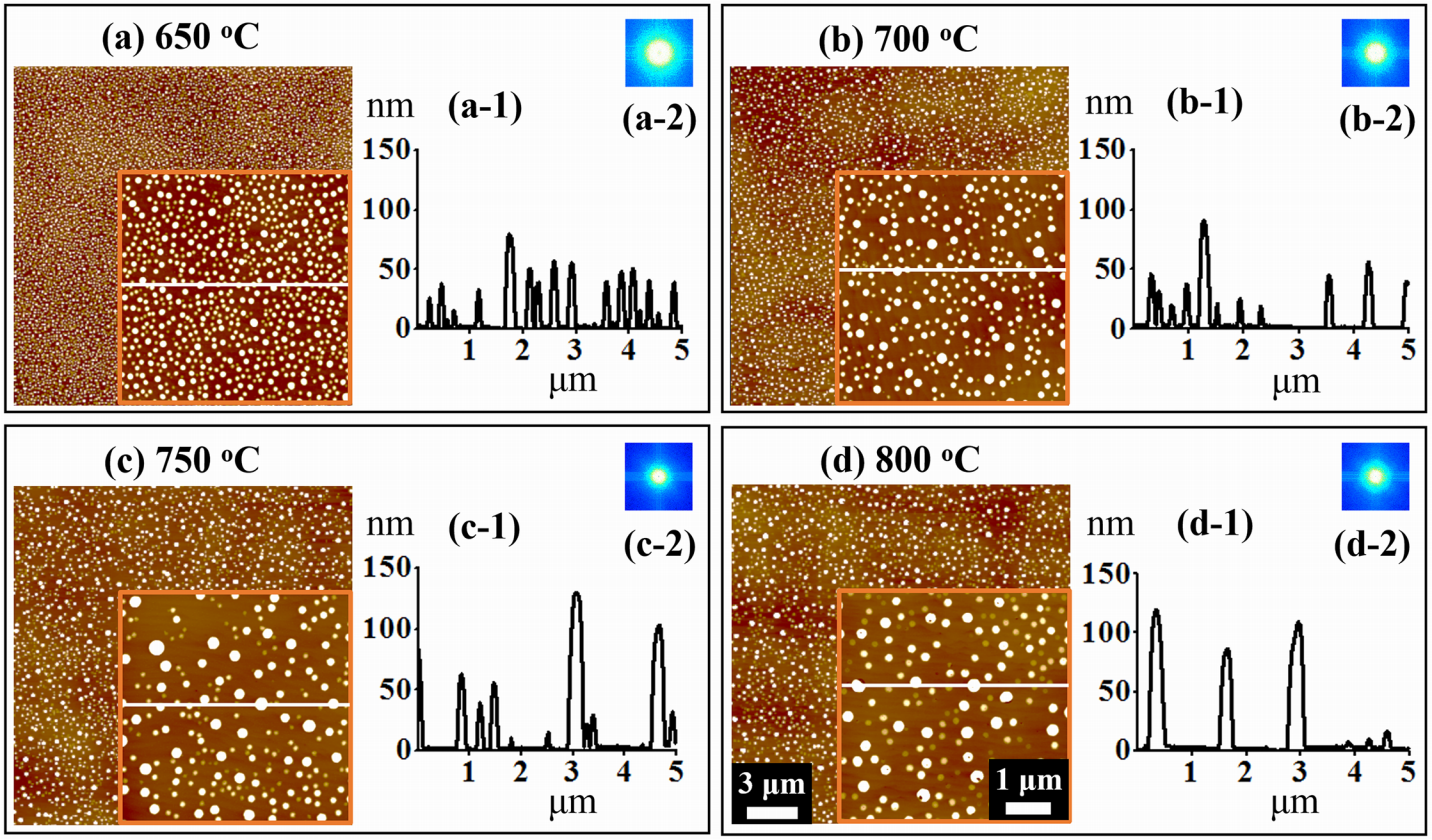
Self-assembled Au NPs fabricated on GaN (0001) with 4 nm Au deposition by the variation of annealing temperature between 650 to 800°C for 300 s. (a)—(d) AFM top-views of 20 × 20 μm^2^ with insets of 5 × 5 μm^2^. (a-1)—(d-1) Cross-sectional surface line-profiles acquired from the white lines. (a-2)—(d-2) FFT power spectra.

**Fig 7:**
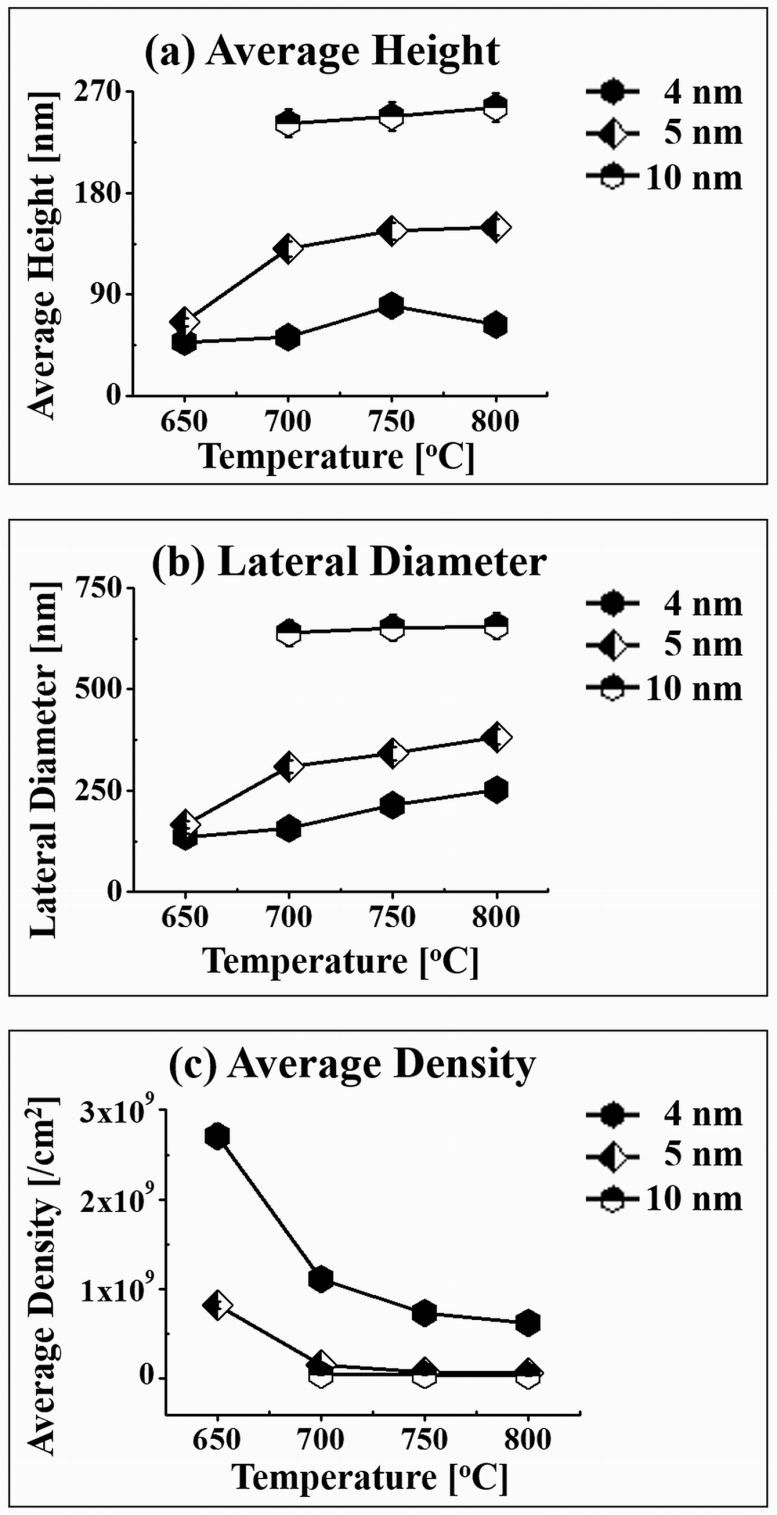
Summary plots of (a) average height, (b) lateral diameter and (c) average density of self-assembled Au NPs fabricated by the variation of annealing temperature (T_a_) along with the variation of Au deposition (DA) on GaN (0001). Error bars: ± 5%.

Fig 6 shows the self-assembled Au NPs fabricated on GaN (0001) with 4 nm of Au deposition by the variation of T_a_ between 650 to 800°C for 300 s. Again, the Au NPs showed a similar behavior to the T_a_ variation and the AH and LD of Au NPs were increased with the increased T_a_ while the AD was gradually decreased. The AH, LD and AD of the self-assembled Au NPs at 650°C were 47.7 nm, 136.1 nm and 2.7 × 10^9^ cm^-2^, respectively. After the 700°C annealing, the AH of Au NPs was increased by 1.08 times to 51.9 nm, the LD was increased by 1.15 times to 156.8 nm while the AD was decreasing by 2.43 times to 1.11 × 10^9^ cm^-2^. At 750°C, the AH and LD were found to be 80.1, 213.9 nm whereas the AD was 7.36 × 10^8^ cm^-2^ and at 800°C, the AH was decreased by 1.27 times to 62.8 nm while the LD was increased by 1.17 times to 252.3 nm and AD was dropped by 1.17 times to 6.24 × 10^8^ cm^-2^. At the T_a_ of 800°C, clearly the height of Au NPs with the DA of 4 nm was decreased while that of 5 and 10 nm DAs were increased as plotted in Fig 7. This may be due to the surface disordering and loss of nitrogen from GaN at higher temperature and appeared more apparently with the lower DA of 4 nm. The AH, LD and AD of the self-assembled NPs of 4, 5 and 10 nm DA samples are plotted in Fig 7 and listed in S1 Table. In general, 4, 5 and 10 nm DAs showed quite similar evolution trends of self-assembled Au NPs, indicating the increased AH and LD and decreased AD as function of T_a_. Overall, with increased Au deposition, relatively large dimension of Au NPs was observed. For example, the 10 nm line is always above the 5 nm line in both AH and LD plots and similarly, the 5 nm line is constantly above the 4. Of course, the density plot is the opposite as the larger NPs should show lower density as described above. As discussed, the diffusion length is determined by the T_a_ and with the increased deposition amount at a specific temperature, more adatoms are available to be absorbed to the initially formed NPs. Being provided with the greater binding energy (E_A_ > E_G_) and sufficient thermal energy, the NPs now can grow larger and with the increased lateral diameter, they can expend the absorption boundary and can grow further until reaching in the equilibrium. Similar results, as one can expect, were observed from the Au NPs on GaAs and Pt NPs on Si substrates. [39–41] But the growth condition was varied accordingly with different substrates for the fabrication of round dome shaped nanoparticles such that Au/GaAs at 550° for 150s and Pt/Si at 800°C for 240s.

## Conclusion

We have successfully demonstrated the fabrication of the self-assembled hexagonal Au voids, nano-clusters and NPs on GaN (0001) through the variation of T_a_ with various deposition amounts of Au. The nucleation of voids and clusters is observed at 400 and 600°C of annealing for 300 s and discussed based on the diffusion limited aggregation model. The size of voids was increased and the density was decreased with the increased T_a_ up to 600°C. When the annealing temperature was raised to 600°C, the self-assembled Au clusters were developed with distinct size. As the T_a_ was further increased above 650°C, the self-assembled Au NPs were successfully demonstrated according to the Volmer-Weber growth model. Overall, with the increased T_a_, the size and density of self-assembled Au NPs followed the same trend on various deposition amounts (4, 5 and 10 nm) of Au: the size was increased while the density was decreased according to the diffusion theory and thermodynamics. Depending upon the deposition amount between 4 to 10 nm with the specific T_a_, the self-assembled Au NPs showed increased dimension with the decreased density. The acquired data were systematically analyzed and discussed with the AFM and SEM images in terms of the top-views and side-views, cross-sectional line-profiles, FFT power spectra and EDS spectra and elemental maps.

## Supporting Information

S1 Fig Raman spectrum of 10 μm-thick GaN (0001) template grown on sapphire measured at room temperature between 150 and 1390 cm^-1^ of Raman shift. The emission was excited by a laser of 532 nm. Each peak is introduced by arrows. The relatively weaker peak at 420 cm^-1^ is due to the sapphire. Furthermore, the E_2_^2^ was observed at 572 cm^-1^ and GaN A_1_(LO) (longitudinal optical) phonon was observed at 738 cm^-1^. The E_2_^2^ is used to monitor the biaxial stresses whereas A_1_(LO) phonon is used to estimate the free carrier concentrations of semiconductors.
(DOCX)

S2 Fig Reflectance spectra of 10 μm-thick GaN template grown on sapphire. (a) UV-Vis-NIR reflectance spectrum over the wavelength between 250 and 1000 nm measured by a CCD. (b) IR reflectance spectrum between 1000 and 2000 nm measured by an InGaAs photodiode. The arrow at 365 nm indicates the cut-off wavelength from where the wave arises or vibrate. Thus, the bandgap can be calculated as 3.397 eV (~3.36 eV similar to the generally known bandgap value of GaN) by using the equation E = h * c / λ. The amplitude and frequency can be dependent upon the thickness of GaN template. The oscillation of 10 μm-thick GaN/sapphire template shows the increased amplitude and frequency as compared with the thinner GaN/sapphire template.
(DOCX)

S3 Fig Three dimensional (3-D) atomic force microscopy (AFM) side-views of Au voids and nano-clusters fabricated on GaN (0001) with 5 nm of Au deposition by the variation of annealing temperature from 400 to 600°C. (a)–(d) Larger scale images of 20 × 20 μm^2^. (a-1)–(d-1) Smaller scale images of 5 × 5 μm^2^.
(DOCX)

S4 Fig Scanning electron microscopy (SEM) images of self-assembled Au nanostructures fabricated on GaN (0001) with 5 nm of Au deposition between 500 and 800°C. (a) Au voids after annealing at 500°C for 300 s. (b) Au nano-clusters after annealing at 600°C. (c)—(f) Evolution of self-assembled Au NPs annealed between 650 and 800°C.
(DOCX)

S5 Fig Energy dispersive X-ray spectroscopy (EDS) maps of Au nano-clusters with the 5 nm of Au deposition annealed at 600°C for 300 s. (a) SEM image. (b) 2-D Phase maps of Au. (c)–(d) 3-D top-views of Au and Ga compositional maps. (e)–(f) EDS spectra of particular region marked with the green and blue square in SEM image. (g)–(h) EDS line-profiles acquired from red lines in specific regions in SEM image.
(DOCX)

S6 Fig 3-D AFM side-views of self-assembled Au NPs with 5 nm of Au deposition on GaN (0001) annealed at (a) 650 (b) 700 (c) 750 and (d) 800°C. (a)–(d) Larger scale images of 20 × 20 μm^2^. (a-1)–(d-1) Smaller scale images of 5 × 5 μm^2^.
(DOCX)

S7 Fig 3-D AFM side-views of self-assembled Au NPs fabricated on GaN (0001) with 10 nm of Au deposition and annealing between 700 and 800°C. (a)–(c) are larger scale images of 20 × 20 μm^2^ whereas (a-1)–(c-1) are enlarged images of 5 × 5 μm^2^.
(DOCX)

S8 Fig SEM images of self-assembled Au NPs on GaN (0001) with 10 nm of Au deposition annealed at (a) 700, (b) 750, and (c) 800°C. (DOCX)

S9 Fig 3-D AFM side-views of self-assembled Au NPs with 4 nm of Au deposition on GaN (0001). Au NPs were annealed between 650 and 800°C. (a)–(d) are 5 × 5 μm2.
(DOCX)

S10 Fig SEM images of Au NPs fabricated on GaN (0001) with of 4 nm Au deposition amount by the variation of annealing temperature between 650 and 800°C. (DOCX)

S11 Fig EDS spectra of (a) 5 nm of Au deposition with 400°C of annealing and (b) 750°C, and (c) 10 nm of Au deposition annealed at 750°C. The Y-axis is counts and the X-axis is the energy of corresponding counts. (a-1)—(c-1) are AFM side-views (5 × 5 μm^2^). (a-2)—(c-2) are enlarged spectra between 1.5 and 2.5 keV, and that of (a-3)–(c-3) are between 9 and 10.5 keV.
(DOCX)

S1 Table Summary of average height (AH), lateral diameter (LD), and average density (AD) of the self-assembled Au NPs fabricated on GaN (0001) with the variation of annealing temperature (T_a_) at various Au deposition amounts (DA). Error range: ± 5%.
(DOCX)
