# Supplementary material for: Systematic Study on the Self-Assembled Hexagonal Au Voids, Nano-Clusters and Nanoparticles on GaN (0001)
Source: PLoS One. 2015 Aug 18;10(8):e0134637. doi: 10.1371/journal.pone.0134637 (PMC4540317; doi:10.1371/journal.pone.0134637)
Supplement: S1 Fig — The emission was excited by a laser of 532 nm. Each peak is introduced by arrows. The relatively weaker peak at 420 cm-1 is due to the sapphire. Furthermore, the E2 2 was observed at 572 cm-1 and GaN A1(LO) (longitudinal optical) phonon was observed at 738 cm-1. The E2 2 is used to monitor the biaxial stresses whereas A1(LO) phonon is used to estimate the free carrier concentrations of semiconductors. (DOCX) [file pone.0134637.s001.docx]

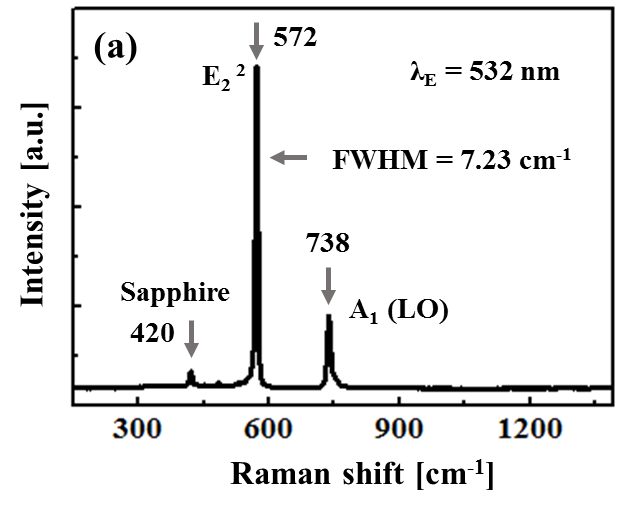


**S1 Fig. Raman spectrum of 10 μm-thick GaN (0001) template grown on sapphire measured at room temperature between 150 and 1390 cm^-1^ of Raman shift.** The emission was excited by a laser of 532 nm. Each peak is introduced by arrows. The relatively weaker peak at 420 cm^-1^ is due to the sapphire.^[1,2]^ Furthermore, the E_2_ ^2^ was observed at 572 cm^-1^ and GaN A_1_(LO) (longitudinal optical) phonon was observed at 738 cm^-1^.^[1-3]^ The E_2_ ^2^ is used to monitor the biaxial stresses whereas A_1_(LO) phonon is used to estimate the free carrier concentrations of semiconductors.

**References**

[1]. H Harima, “Properties of GaN and related compounds studied by means of Raman scattering” *Journal of Physics: Condensed Matter*, **14**, R967–R993 (2002).

[2]. R. Fornari, M. Bosi, D. Bersani, G. Attolini, P. P. Lottici and C. Pelosi, “Characterization of HVPE GaN layers by atomic force microscopy and Raman spectroscopy” *Semiconductor science and technology*, **16**, 776 (2001).

[3]. H. Y. Kim, J. A. Freitas and J. Kim, “Penetration effects of high-energy protons in GaN: a micro-Raman spectroscopy study” *Electrochemical and Solid-State Letters*, **14**, H5-H8 (2011).
