# Supplementary material for: Systematic Study on the Self-Assembled Hexagonal Au Voids, Nano-Clusters and Nanoparticles on GaN (0001)
Source: PLoS One. 2015 Aug 18;10(8):e0134637. doi: 10.1371/journal.pone.0134637 (PMC4540317; doi:10.1371/journal.pone.0134637)
Supplement: S2 Fig — (a) UV-Vis-NIR reflectance spectrum over the wavelength between 250 and 1000 nm measured by a CCD. (b) IR reflectance spectrum between 1000 and 2000 nm measured by an InGaAs photodiode. The arrow at 365 nm indicates the cut-off wavelength from where the wave arises or vibrate. Thus, the bandgap can be calculated as 3.397 eV (~3.36 eV similar to the generally known bandgap value of GaN) by using the equation E = h * c / λ. The amplitude and frequency can be dependent upon the thickness of GaN template. The oscillation of 10 μm-thick GaN/sapphire template shows the increased amplitude and frequency as compared with the thinner GaN/sapphire template. (DOCX) [file pone.0134637.s002.docx]

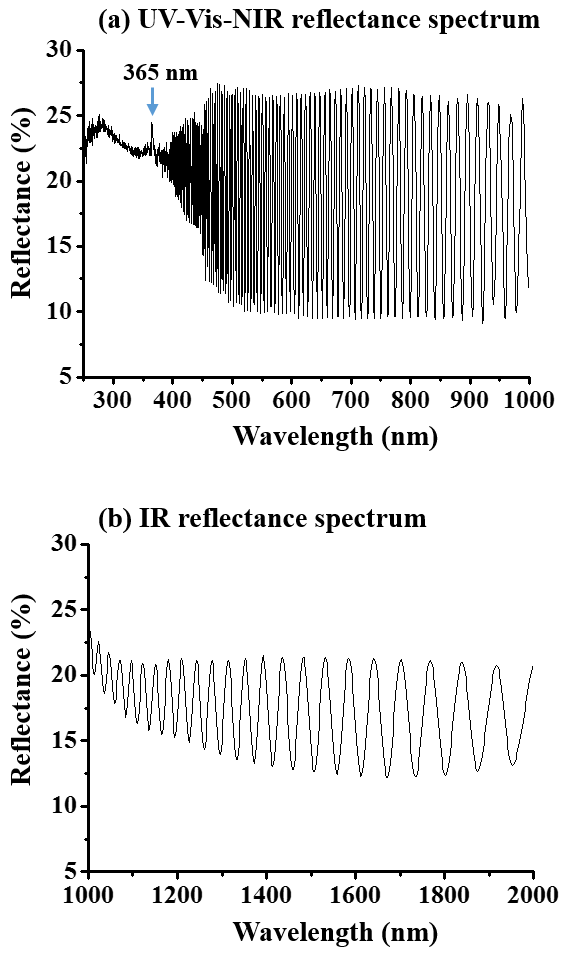


**S2 Fig. Reflectance spectra of 10 μm-thick GaN template grown on sapphire. (a) UV-Vis-NIR reflectance spectrum over the wavelength between 250 and 1000 nm measured by a CCD.** (b) IR reflectance spectrum between 1000 and 2000 nm measured by an InGaAs photodiode. The arrow at 365 nm indicates the cut-off wavelength from where the wave arises or vibrate. Thus, the bandgap can be calculated as 3.397 eV (~3.36 eV similar to the generally known bandgap value of GaN) by using the equation E = h * c / λ. The amplitude and frequency can be dependent upon the thickness of GaN template. ^[1]^ The oscillation of 10 μm-thick GaN/sapphire template shows the increased amplitude and frequency as compared with the thinner GaN/sapphire template.^[1]^

**Reference**

[1]. C. X.Lian, X. Y. Li and J. Liu, “Optical anisotropy of wurtzite GaN on sapphire characterized by spectroscopic ellipsometry” *Semiconductor science and technology*, **19**, 417 (2004).
