# Supplementary material for: Systematic Study on the Self-Assembled Hexagonal Au Voids, Nano-Clusters and Nanoparticles on GaN (0001)
Source: PLoS One. 2015 Aug 18;10(8):e0134637. doi: 10.1371/journal.pone.0134637 (PMC4540317; doi:10.1371/journal.pone.0134637)
Supplement: S3 Fig — (a)–(d) Larger scale images of 20 × 20 μm2. (a-1)–(d-1) Smaller scale images of 5 × 5 μm2. (DOCX) [file pone.0134637.s003.docx]

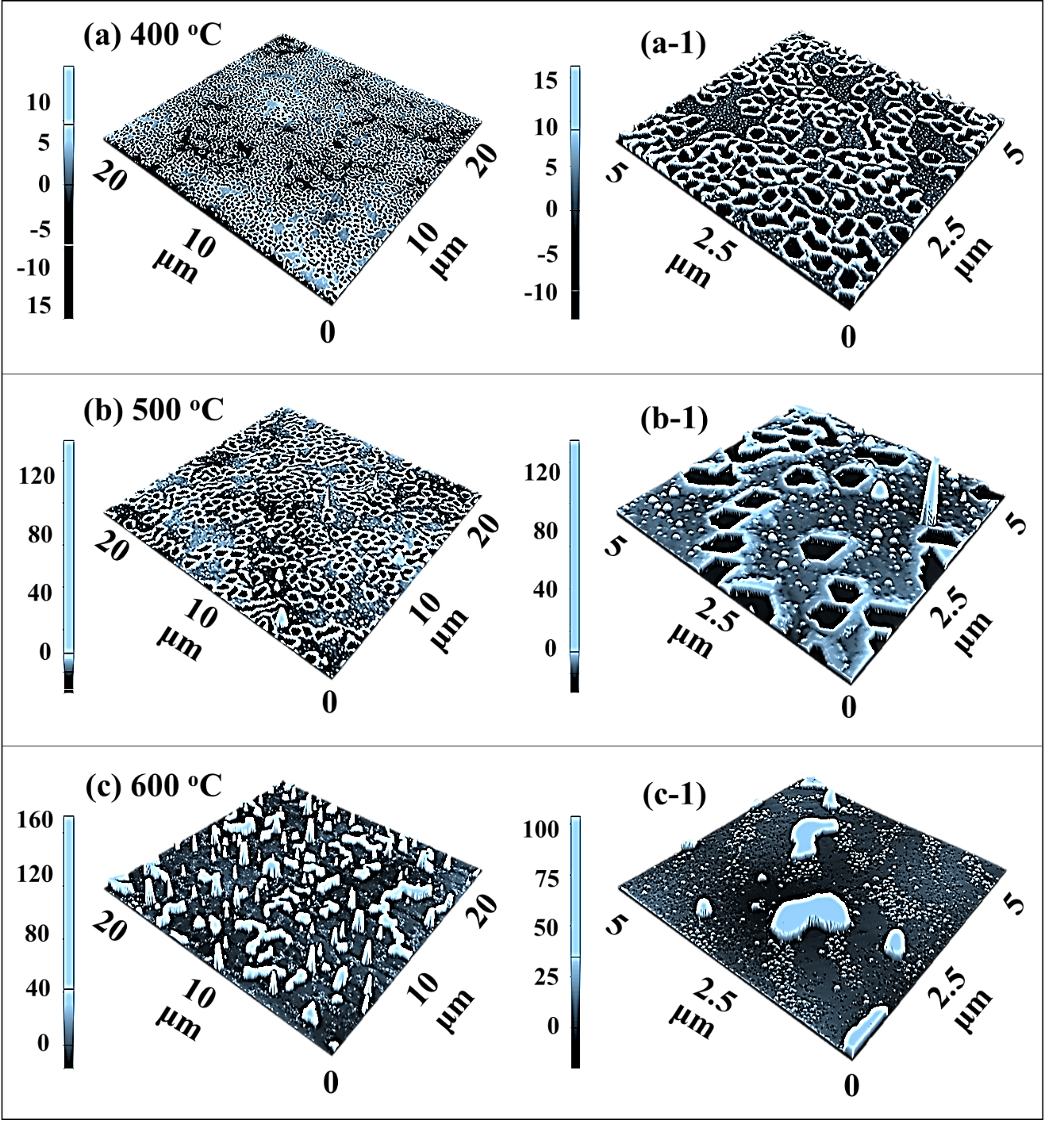


**S3 Fig. Three dimensional (3-D) atomic force microscopy (AFM) side-views of Au voids and nano-clusters fabricated on GaN (0001) with 5 nm of Au deposition by the variation of annealing temperature from 400 to 600 ^o^C.** (a) – (d) Larger scale images of 20 × 20 μm^2^. (a-1) – (d-1) Smaller scale images of 5 × 5 μm^2^.
