# Supplementary material for: Systematic Study on the Self-Assembled Hexagonal Au Voids, Nano-Clusters and Nanoparticles on GaN (0001)
Source: PLoS One. 2015 Aug 18;10(8):e0134637. doi: 10.1371/journal.pone.0134637 (PMC4540317; doi:10.1371/journal.pone.0134637)
Supplement: S4 Fig — (a) Au voids after annealing at 500°C for 300 s. (b) Au nano-clusters after annealing at 600°C. (c)—(f) Evolution of self-assembled Au NPs annealed between 650 and 800°C. (DOCX) [file pone.0134637.s004.docx]

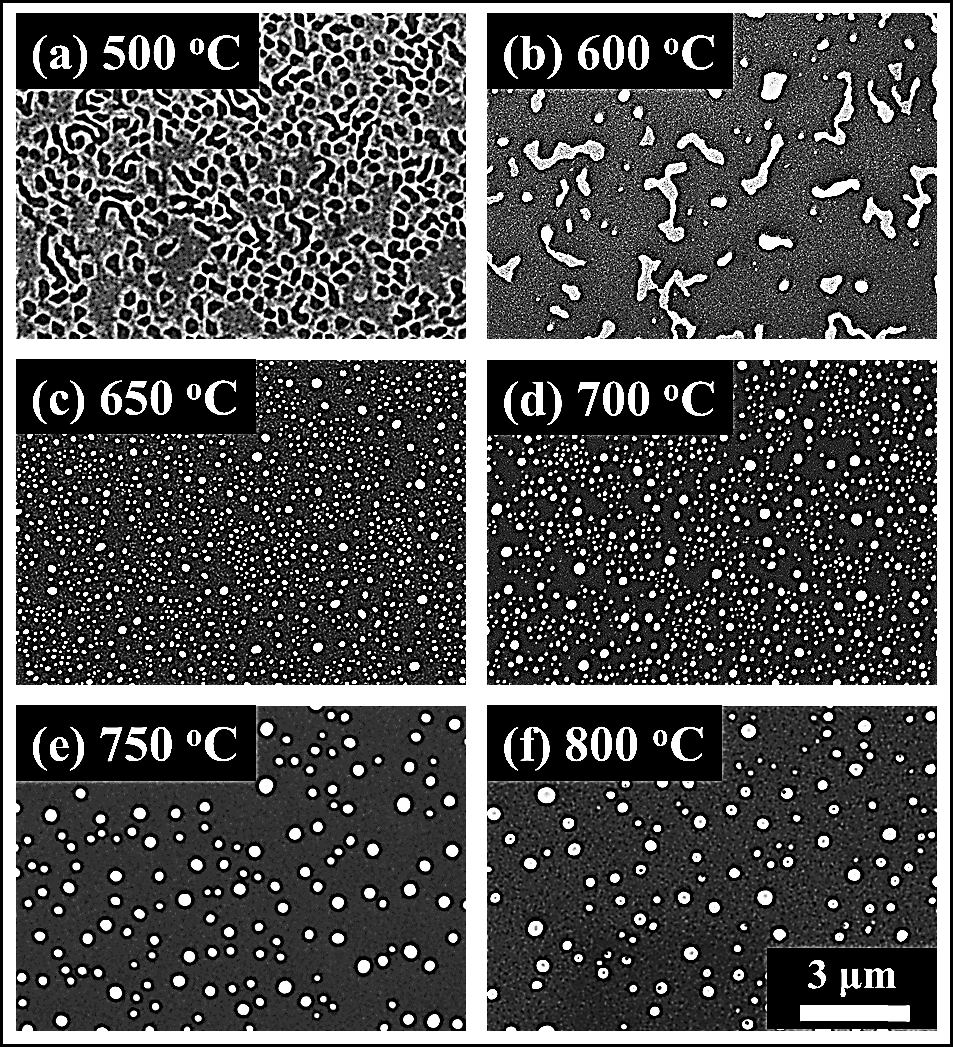


**S4 Fig. Scanning electron microscopy (SEM) images of self-assembled Au nanostructures fabricated on GaN (0001) with 5 nm of Au deposition between 500 and 800 ^o^C.** (a) Au voids after annealing at 500 ^o^C for 300 s. (b) Au nano-clusters after annealing at 600 ^o^C. (c) - (f) Evolution of self-assembled Au NPs annealed between 650 and 800 ^o^C.
