# Supplementary material for: Systematic Study on the Self-Assembled Hexagonal Au Voids, Nano-Clusters and Nanoparticles on GaN (0001)
Source: PLoS One. 2015 Aug 18;10(8):e0134637. doi: 10.1371/journal.pone.0134637 (PMC4540317; doi:10.1371/journal.pone.0134637)
Supplement: S5 Fig — (a) SEM image. (b) 2-D Phase maps of Au. (c)–(d) 3-D top-views of Au and Ga compositional maps. (e)–(f) EDS spectra of particular region marked with the green and blue square in SEM image. (g)–(h) EDS line-profiles acquired from red lines in specific regions in SEM image. (DOCX) [file pone.0134637.s005.docx]

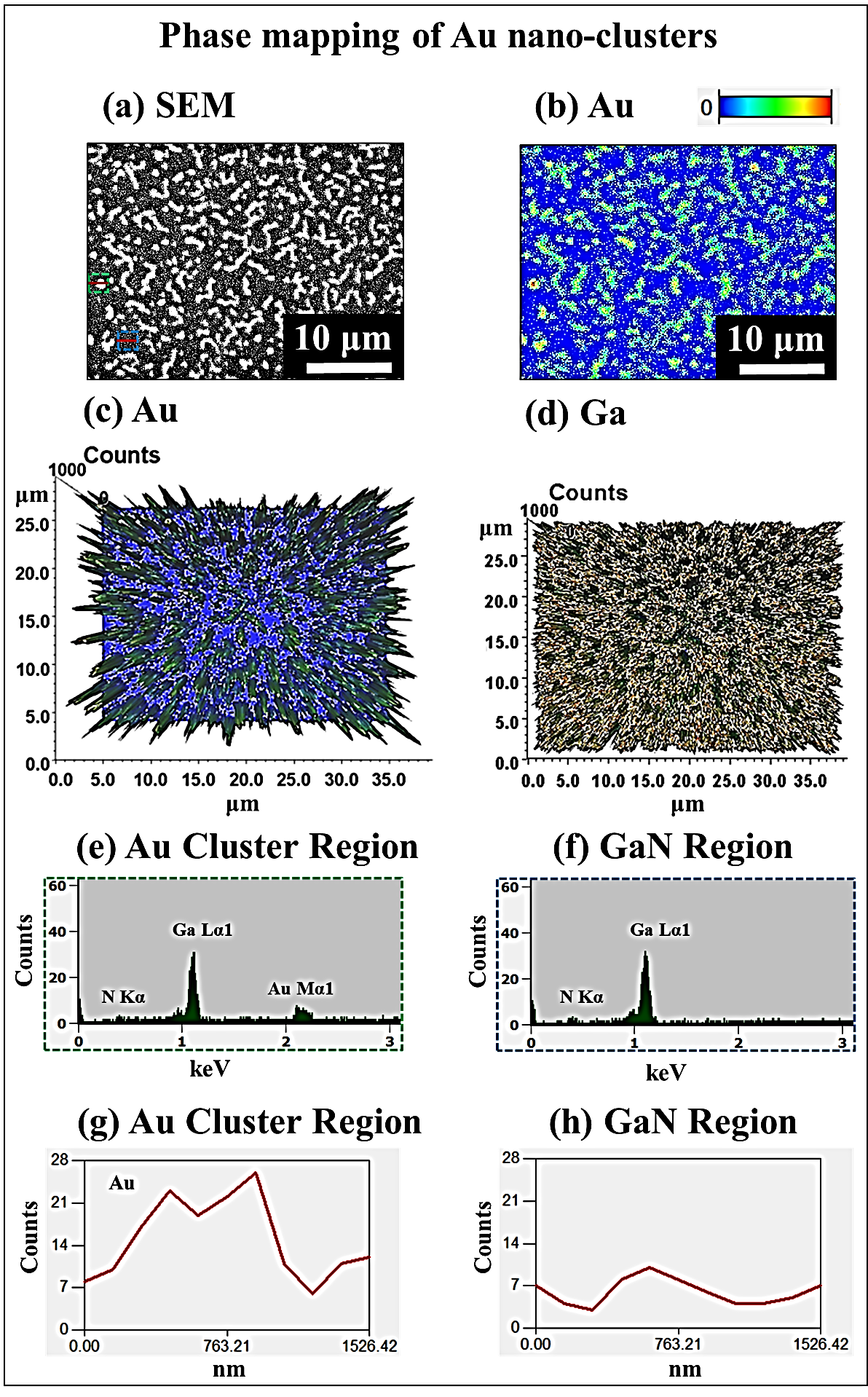


**S5 Fig. Energy dispersive X-ray spectroscopy (EDS) maps of Au nano-clusters with the 5 nm of Au deposition annealed at 600 ^o^C for 300 s.** (a) SEM image. (b) 2-D Phase maps of Au. (c) – (d) 3-D top-views of Au and Ga compositional maps. (e) – (f) EDS spectra of particular region marked with the green and blue square in SEM image. (g) – (h) EDS line-profiles acquired from red lines in specific regions in SEM image.

S5 Fig shows the energy dispersive X-ray spectroscopy (EDS) phase maps of Au nano-clusters with 5 nm of Au deposition annealed at 600 ^o^C for 300 s. The EDS has been utilized to map the regions of particular elements. The SEM image in Fig. S5(a) shows the morphology of the Au clusters and Fig. S5(b) shows the 2-D phase map of Au. As clearly seen in phase map, the higher concentration of Au was clearly shown with the yellow regions whereas there is no presence of Au on blue regions. The Au clusters were clearly evidenced with higher counts as shown by the 3-D top view of the compositional map in Figs. S5(c). In 3-D phase maps of Ga, the holes corresponds to the Au clusters and the remaining parts are covered with Ga as shown in Fig. S5(d). The concentration of Au in specific region of Au cluster denoted by a green square in Fig. S5(a) shows the presence of the Au peak while the region marked by a blue square without Au cluster does not show any Au as clearly shown by the EDS spectra in Figs. S5(e) - S5(f). Similarly, the line profiles on corresponding regions also evidenced the high counts of Au in cluster region as shown in Figs. S5(g) - S5(h).
