# Supplementary material for: Systematic Study on the Self-Assembled Hexagonal Au Voids, Nano-Clusters and Nanoparticles on GaN (0001)
Source: PLoS One. 2015 Aug 18;10(8):e0134637. doi: 10.1371/journal.pone.0134637 (PMC4540317; doi:10.1371/journal.pone.0134637)
Supplement: S6 Fig — (a)–(d) Larger scale images of 20 × 20 μm2. (a-1)–(d-1) Smaller scale images of 5 × 5 μm2. (DOCX) [file pone.0134637.s006.docx]

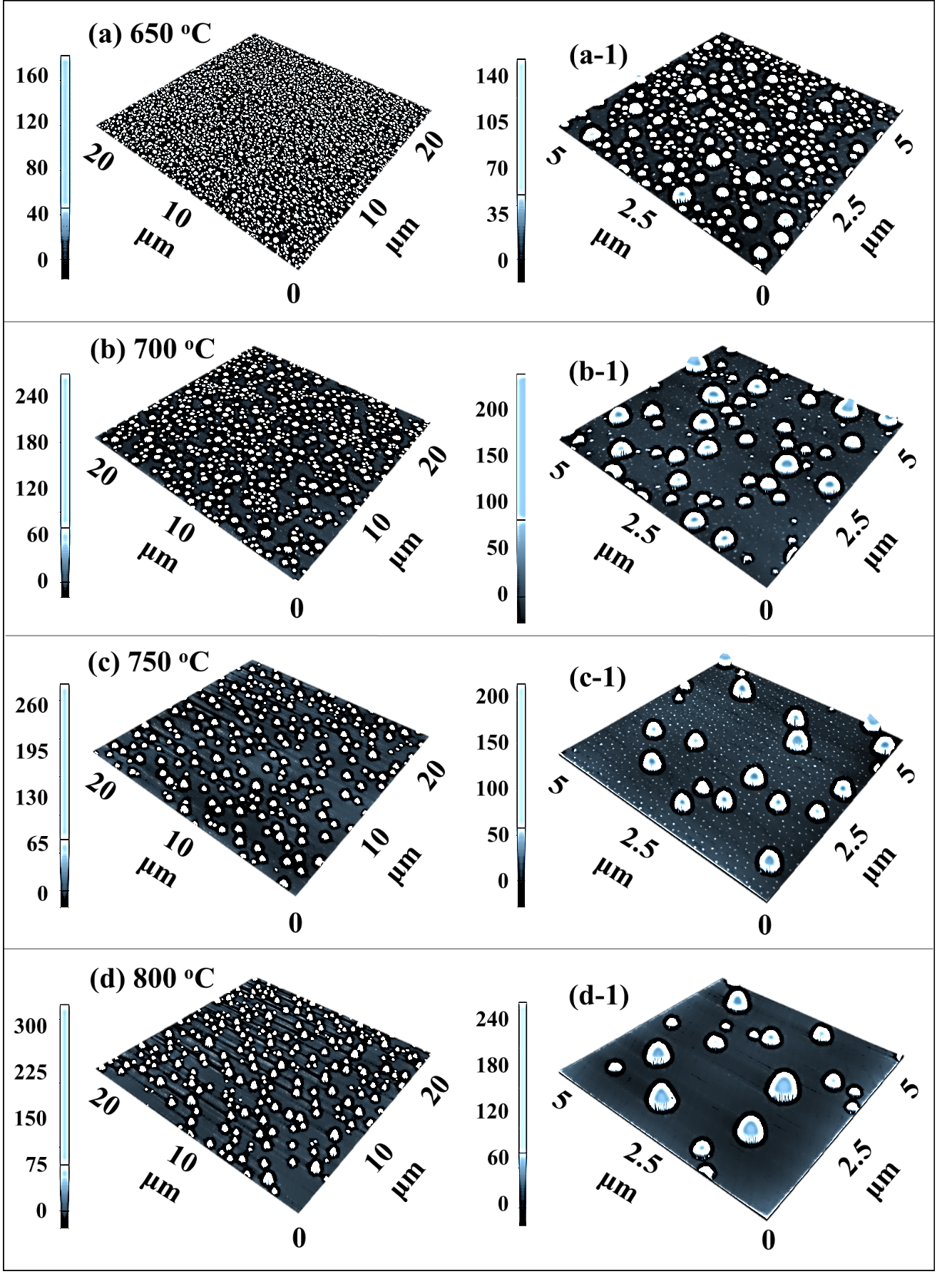


**S6 Fig. 3-D AFM side-views of self-assembled Au NPs with 5 nm of Au deposition on GaN (0001) annealed at (a) 650 (b) 700 (c) 750 and (d) 800 ^o^C**. (a) – (d) Larger scale images of 20 × 20 μm^2^. (a-1) – (d-1) Smaller scale images of 5 × 5 μm^2^.
