# Supplementary material for: Systematic Study on the Self-Assembled Hexagonal Au Voids, Nano-Clusters and Nanoparticles on GaN (0001)
Source: PLoS One. 2015 Aug 18;10(8):e0134637. doi: 10.1371/journal.pone.0134637 (PMC4540317; doi:10.1371/journal.pone.0134637)
Supplement: S7 Fig — (a)–(c) are larger scale images of 20 × 20 μm2 whereas (a-1)–(c-1) are enlarged images of 5 × 5 μm2. (DOCX) [file pone.0134637.s007.docx]

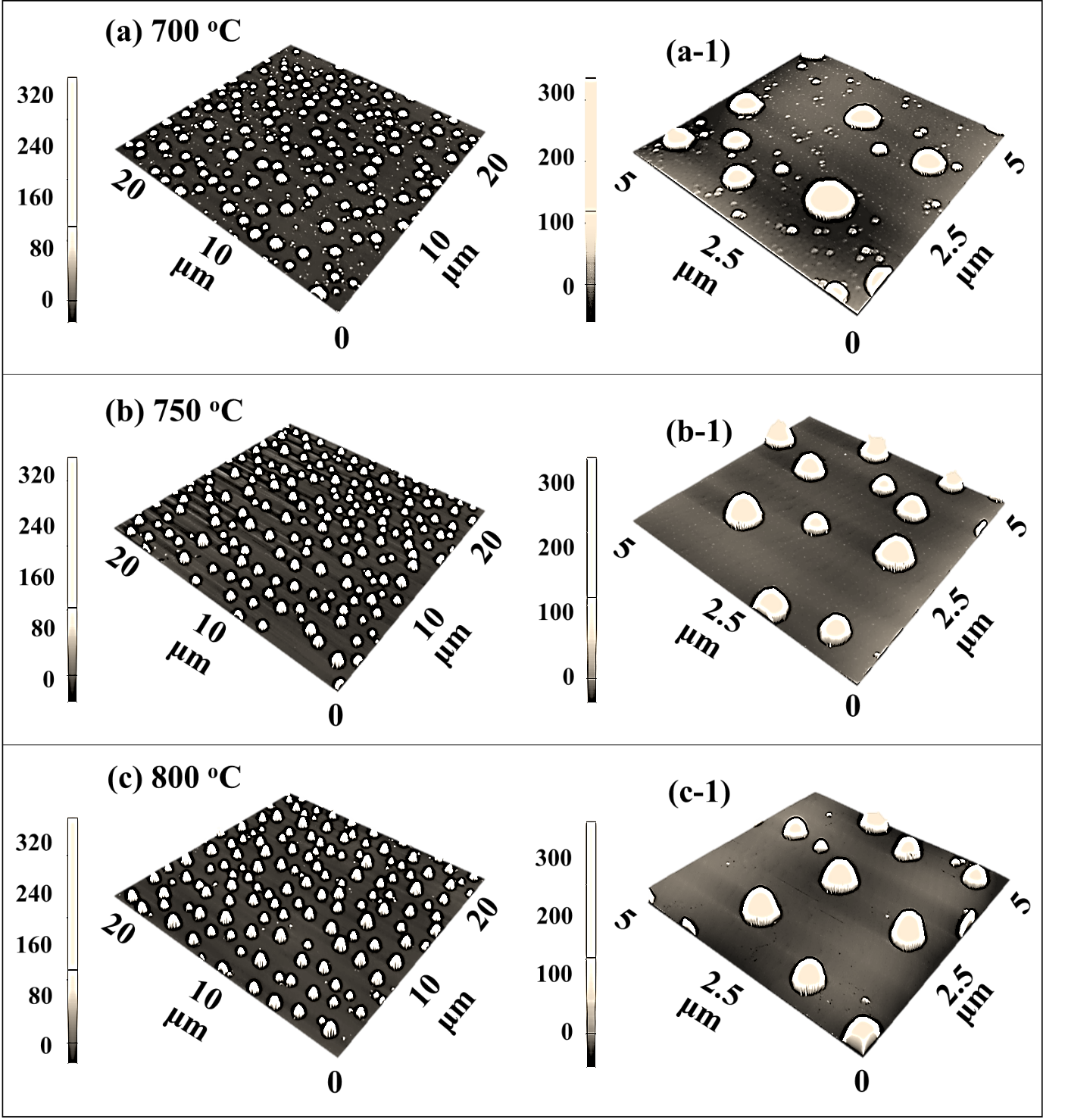


**S7 Fig. 3-D AFM side-views of self-assembled Au NPs fabricated on GaN (0001) with 10 nm of Au deposition and annealing between 700 and 800 ^o^C.** (a) – (c) are larger scale images of 20 × 20 μm^2^ whereas (a-1) – (c-1) are enlarged images of 5 × 5 μm^2^.
