# Supplementary figures and images for: Systematic Study on the Self-Assembled Hexagonal Au Voids, Nano-Clusters and Nanoparticles on GaN (0001)
Source: PLoS One. 2015 Aug 18;10(8):e0134637. doi: 10.1371/journal.pone.0134637 (PMC4540317; doi:10.1371/journal.pone.0134637)

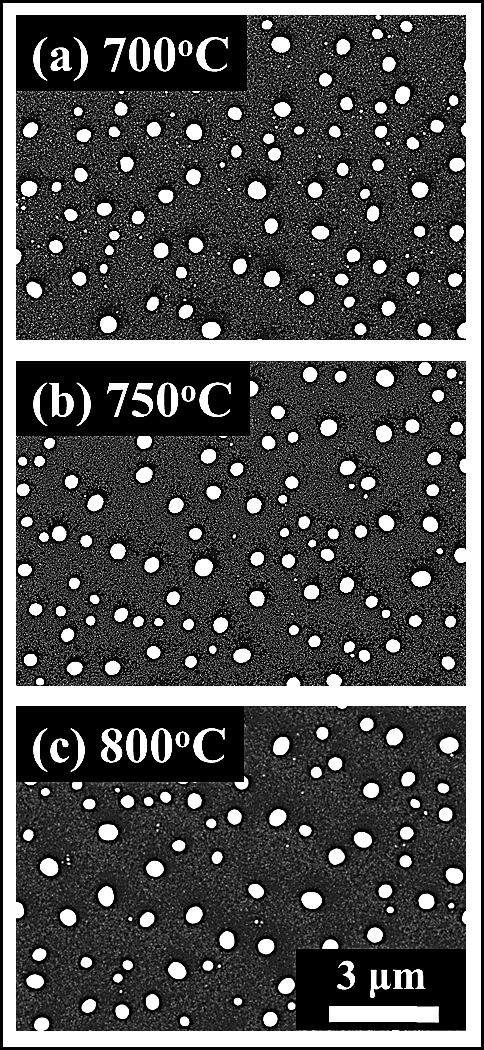


**S8 Fig.** SEM images of self-assembled Au NPs on GaN (0001) with10 nm of Au deposition annealed at (a) 700, (b) 750, and (c) 800 oC.

Supplement: S8 Fig — (DOCX) [file pone.0134637.s008.docx]
