# Supplementary material for: Systematic Study on the Self-Assembled Hexagonal Au Voids, Nano-Clusters and Nanoparticles on GaN (0001)
Source: PLoS One. 2015 Aug 18;10(8):e0134637. doi: 10.1371/journal.pone.0134637 (PMC4540317; doi:10.1371/journal.pone.0134637)
Supplement: S9 Fig — Au NPs were annealed between 650 and 800°C. (a)–(d) are 5 × 5 μm2. (DOCX) [file pone.0134637.s009.docx]

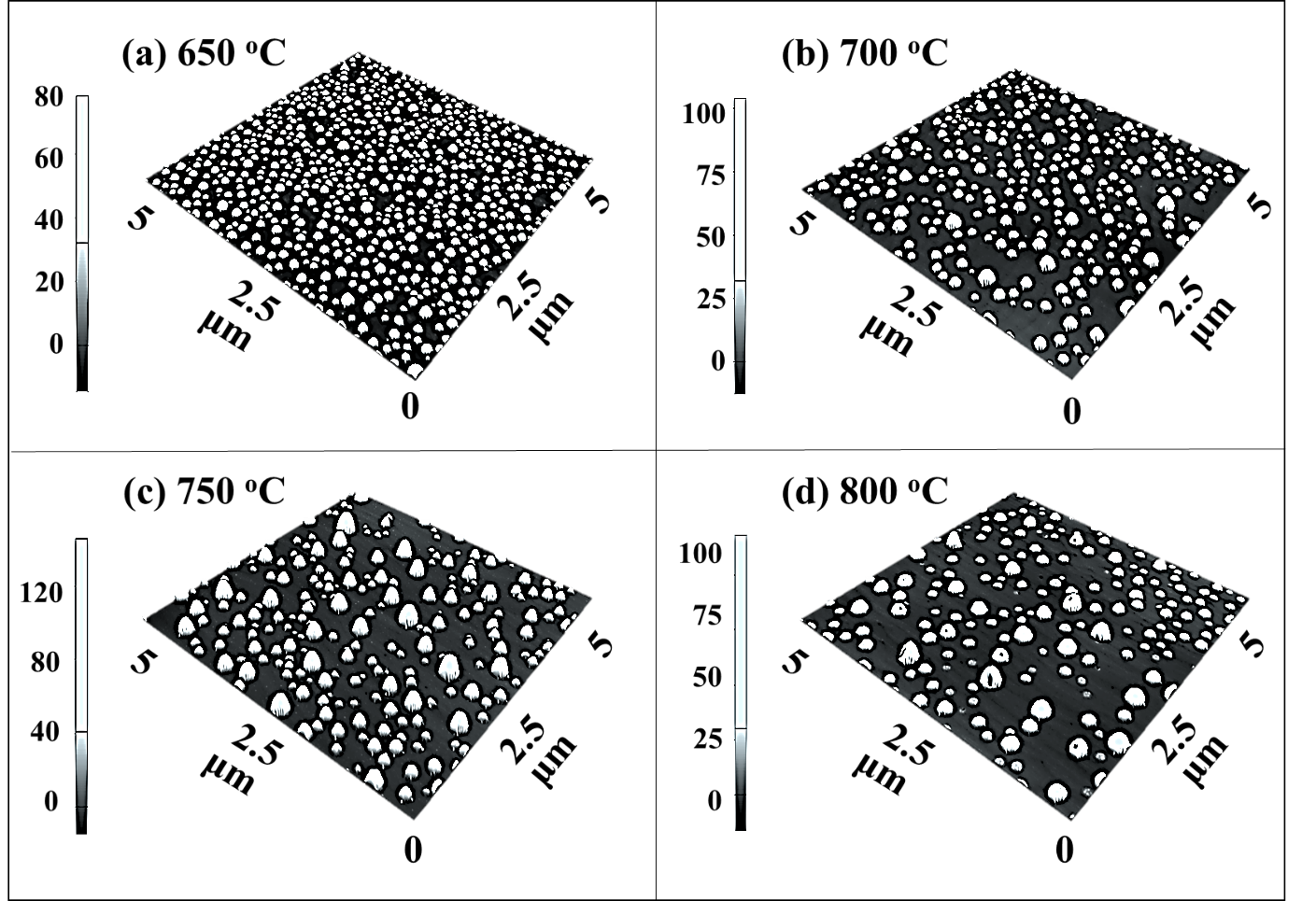


**S9 Fig.** 3-D AFM side-views of self-assembled Au NPs with 4 nm of Au deposition on GaN (0001). Au NPs were annealed between 650 and 800 ^o^C. (a) – (d) are 5 × 5 μm^2^.
