# Supplementary material for: Systematic Study on the Self-Assembled Hexagonal Au Voids, Nano-Clusters and Nanoparticles on GaN (0001)
Source: PLoS One. 2015 Aug 18;10(8):e0134637. doi: 10.1371/journal.pone.0134637 (PMC4540317; doi:10.1371/journal.pone.0134637)
Supplement: S10 Fig — (DOCX) [file pone.0134637.s010.docx]

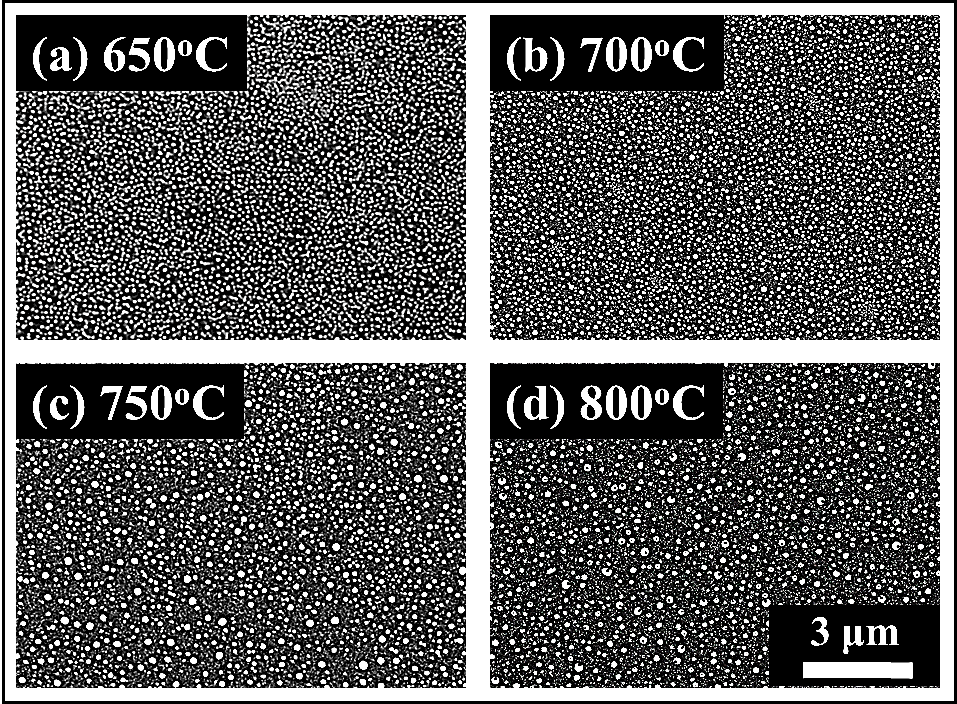


**S10 Fig.** SEM images of Au NPs fabricated on GaN (0001) with of 4 nm Au deposition amount by the variation of annealing temperature between 650 and 800 ^o^C.
