# Supplementary material for: Systematic Study on the Self-Assembled Hexagonal Au Voids, Nano-Clusters and Nanoparticles on GaN (0001)
Source: PLoS One. 2015 Aug 18;10(8):e0134637. doi: 10.1371/journal.pone.0134637 (PMC4540317; doi:10.1371/journal.pone.0134637)
Supplement: S11 Fig — The Y-axis is counts and the X-axis is the energy of corresponding counts. (a-1)—(c-1) are AFM side-views (5 × 5 μm2). (a-2)—(c-2) are enlarged spectra between 1.5 and 2.5 keV, and that of (a-3)–(c-3) are between 9 and 10.5 keV. (DOCX) [file pone.0134637.s011.docx]

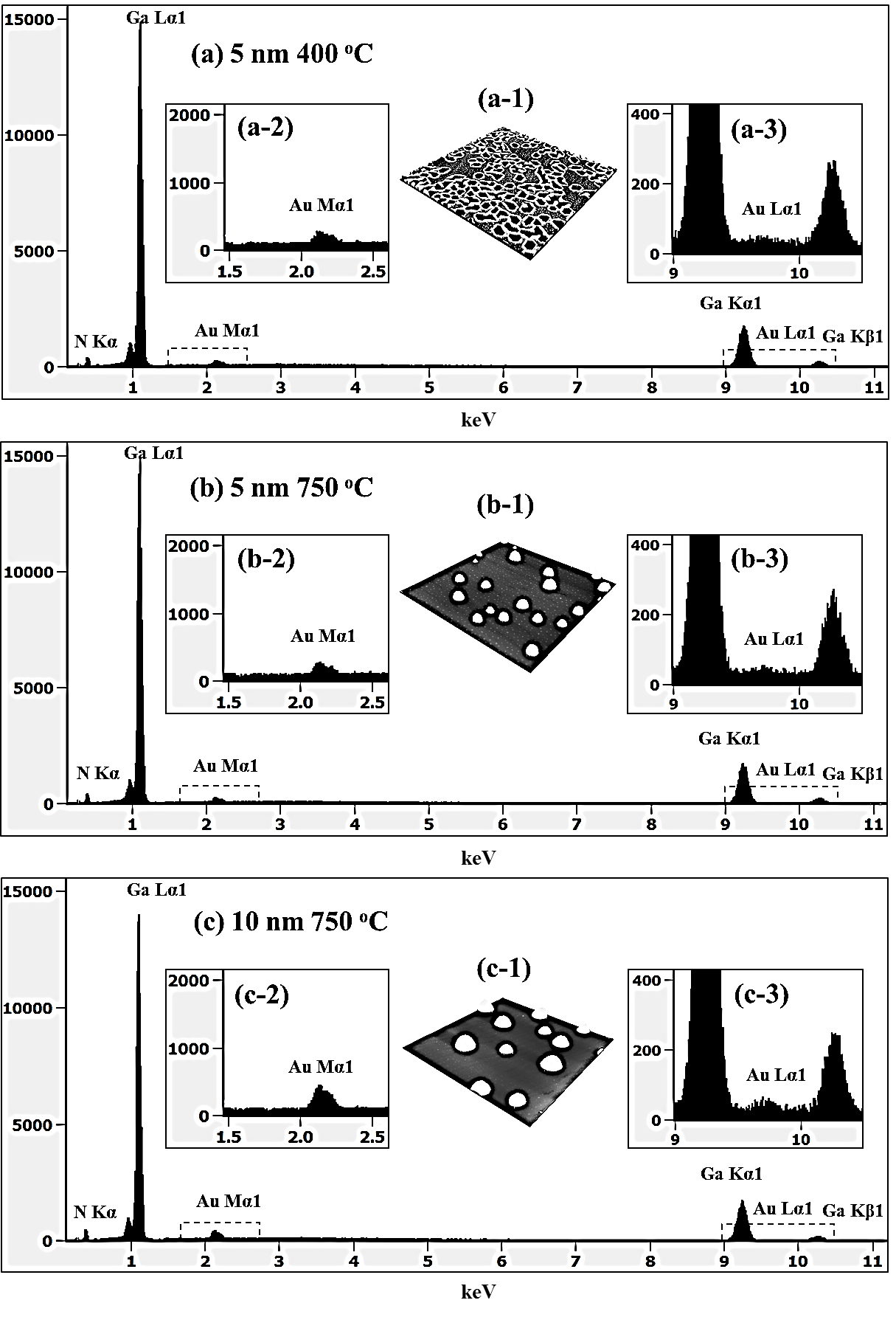


**S11 Fig.** **EDS spectra of (a) 5 nm of Au deposition with 400 ^o^C of annealing and (b) 750 ^o^C, and (c) 10 nm of Au deposition annealed at 750 ^o^C.** The Y-axis is counts and the X-axis is the energy of corresponding counts. (a-1) - (c-1) are AFM side-views (5 × 5 μm^2^). (a-2) - (c-2) are enlarged spectra between 1.5 and 2.5 keV, and that of (a-3) – (c-3) are between 9 and 10.5 keV.

Figure S11 shows the EDS spectra of 5 nm Au deposition annealed at 400 ^o^C in Fig. S11(a) and at 750 ^o^C in Fig. S11(b). Fig. S11(c) shows the spectra with the 10 nm Au deposition at 750 ^o^C for 300 s. The Y-axis shows the counts and the X-axis indicates the energy level of corresponding counts. The enlarged spectra between 1.5 and 2.5 keV show the peak count of Au Mα1 in Figs. S11(a-2) – S11(c-2) and similarly the enlarged spectra between 9 and 10.5 keV show the peak count of Au Lα1 in Figs. S11(a-3) – S11(c-3). As shown in Fig. S11(a) – S11(b), with the variation of T_a_ of 400 and 750 ^o^C with 5 nm deposition, although the AFM side-view of corresponding samples show the distinctive morphologies in Figs. S11(a-1) and S11(b-1), the Au Mα1 peak at 2.136 keV and Au Lα1peak at 9.741 keV were nearly identical. But when the Au deposition amount was raised to 10 nm keeping T_a_ fixed 750 ^o^C, comparatively higher Au Mα1 peak at 2.136 keV was observed as shown in Fig. S11(c) likely due to the increased interaction volume of Au atoms with X-ray. When Au deposition amount was increased to 10 nm, Au Mα1 peak showed approximately
