# Supplementary material for: Systematic Study on the Self-Assembled Hexagonal Au Voids, Nano-Clusters and Nanoparticles on GaN (0001)
Source: PLoS One. 2015 Aug 18;10(8):e0134637. doi: 10.1371/journal.pone.0134637 (PMC4540317; doi:10.1371/journal.pone.0134637)
Supplement: S1 Table — Error range: ± 5%. (DOCX) [file pone.0134637.s012.docx]

|  | **T_a_**  **[ ^o^C]**  **DA**  **[nm]** | **650** | **700** | **750** | **800** |
| --- | --- | --- | --- | --- | --- |
| **AH**  **[nm]** | 4 | 47.7 | 51.9 | 80.1 | 62.8 |
|  | 5 | 65.2 | 130.2 | 146.2 | 149.6 |
|  | 10 | - | 242.1 | 248.3 | 256.2 |
| **LD**  **[nm]** | 4 | 136.1 | 156.8 | 213.9 | 252.3 |
|  | 5 | 165.4 | 309.1 | 341.4 | 382.4 |
|  | 10 | - | 638.9 | 651.1 | 656.2 |
| **AD**  **[×10^6^/cm^2^]** | 4 | 2708 | 1112 | 736 | 624 |
|  | 5 | 820 | 156 | 72 | 60 |
|  | 10 | - | 45.25 | 42 | 32.75 |

**S1 Table. Summary of average height (AH), lateral diameter (LD), and average density (AD) of the self-assembled Au NPs fabricated on GaN (0001) with the variation of annealing temperature (T_a_) at various Au deposition amounts (DA).** Error range: ± 5%.
